# Trench Foot

**Published:** 1918-12

**Authors:** J. Cottet


VOL. II, NO. 5 PARIS DECEMBER 1918

### WAR MEDICINE

Published by the American Red Cross Society in France
for the
Medical Officers of the American Expeditionary Forces

### RESEARCH SOCIETY REPORTS

THE EIGHTH SESSION OF THE RESEARCH SOCIETY OF THE AMERICAN RED CROSS IN FRANCE

October 4 and 5, 1918, at the Hotel Continental, Paris.

(Report continued from November number.)

The program of these meetings and the reports of the papers on "Streptococcus Infections of the Respiratory Tract" appeared in the November number of *War Medicine*.

### TRENCH FOOT

**Trench Foot (Etiology — Pathology — Symptomatology).** By J. COTTET.

This name is given to a set of vaso-motor, nervous, and trophic complications of the feet of soldiers immobilized in the trenches. These complications may vary from a simple swelling to a more or less serious necrotic process, all of which are accompanied by acute and persistent pain. The temperature is normal as long as there are no infectious complications.

The causes of these accidents seldom vary. They are witnessed in the infantry where men have to remain in trenches and shell holes motionless, with their feet in water or mud, for a period of from two to five days. The man begins to feel a painful cold and numbing sensation. Then he experiences a prickling and burning sensation extending to the legs, which becomes strong enough to prevent him from walking or standing up. Sometimes the disease breaks out only when the man gets back to his cantonment. When he takes his shoes off, his feet are painful and swollen. The swelling increases, and he cannot put his shoes on again.

The immersion of the lower extremities in water or mud, prolonged immobilization in a vertical or sitting down position, the impossibility of taking one's shoes off, are the usual, but not constant, etiological circumstances on which such a condition depend. The disease is seen in men of all ages. Such local complications as varices, flat feet, etc., do not seem to play an important part. Certain men have been able to avoid the disease by taking good care of their shoes and feet, and by remaining in a stationary position as little as possible. As a rule both feet are affected. However, one may be more so than the other. Sometimes accidents of the same kind, but in a milder form, affect the hands.

The etiological importance of humidity explains the frequency of this affection in winter, spring, and autumn, during the period of rain and thaw. It occurs very seldom in dry, cold weather.

These accidents, which were very frequent during the first winters of the war, and caused terrible wastage, occurred less often as the trenches were better organized and dried, as men were more frequently relieved from first line duty, and as attention was drawn to the care of the feet and shoes.

In the milder forms, which are by far the most frequent, the lesions are superficial and do not go beyond the hypoderma. As a rule there is a painful swelling of the foot, which may extend up the leg. The skin which is drawn, shiny, and often erythematous and warm, is sometimes marked with small dots, due to hemorrhage. In many of the cases there are no other apparent lesions. Very often there are blisters, which vary in size. Their shape is irregular, and they may contain serum or blood. Finally, even in the mild forms, the skin is mottled and in certain points, especially at the metatarso-phalangean articulation of the big toe, it has a purple color.

These objective manifestations diminish and disappear rapidly, as a rule at the end of a week, under the influence of rest and protective dressings. At the decline of this infection an epidemic desquamation occurs, which can be compared to that of scarlet fever. This desquamation, which occurs even in the lightest cases, may be seen on the hands, which proves that the latter can be affected by the same accidents, although more rarely and to a lesser degree.

The serious forms, which fortunately occur rather seldom, are characterized by the more or less deep mortification of the tissues of the foot. The latter is cold, insensible, mottled with faint purple blotches, and is covered with blisters containing blood. This picture reminds one of the accidents which are seen in gangrene by arterial obliteration. The beats of the dorsalis pedis artery cannot be perceived, and if an oscillometric study was made of the arterial beats at the level of the instep, a great diminution of amplitude would doubtless be witnessed.

The evolution of the serious forms is marked by the formation and elimination of scabs, and even sequestrae, when the skeleton is injured. This process of mortification occurs under the form of either dry or purulent gangrene. It ends up with the loss of substance, which as a rule involves only the toes, but which may go as far as to affect a part, or even the whole, of the foot. Sometimes the reparation is obtained by a spontaneous process of elimination of the scabs. Sometimes it is necessary to correct and to complete this process by a surgical intervention. The cicatrization takes a very long time, and the cicatrix has a tendency to ulcerate in proportion to the diminution of vitality of the tissues. Among the objective symptoms are the complications of cutaneous sensibility. They exist in the milder forms as well as in the serious forms. Some are local; others are general,

The former, which occur in the acute stage, break out exclusively in the feet. These are : anesthesic areas, often coinciding with hyperesthesic areas.

Besides those local complications, one may observe objective modifications of cutaneous sensibility, which affect the limbs and often the nose and ears. This is a hypesthesia, which could be more rightly called hypalgesia, for it bears more on painful and thermic, rather than tactile, sensations. It affects symmetrically the four limbs. The superior limit follows a plan which is perpendicular to the axis of the limb, which corresponds to the hip joint for the inferior limbs, and to the shoulder for the superior limbs. As a rule it is less marked in the thigh and arm than in the leg and forearm. Its intensity varies greatly. In some cases it is hardly perceptible, whereas in others the patient may endure without any pain the pricking of a needle through the tegument. A remarkable particularity of this hypesthesia, which is unexplained, is the parts which it involves. It affects only the back of the hand, and only the sole of the foot.

As a matter of fact, this is a sensitive syndrome, as well as cutaneous, peripheric, terminal, symetric and segmentary in its distribution, affecting neither the nervous nor radicular areas. It is evidently caused by vasomotor perturbation under the influence of the sympathetic nerve, which I have proposed to call " syndrome of seroparesthesia with frigosis ", on account of the important part which cold plays in the determination of such phenomena. This sensitive syndrome, which is constant in cases which have, or have had frozen feet, is frequently witnessed in men who have to undergo frequent changes in weather, and who are exposed to intense cold. The modifications of reflectivity involve only the plantar cutaneous reflex, which is very often diminished or abolished, owing to the plantar hypesthesia. Very seldom the achillian reflexes, and, more seldom yet, the patellar reflexes are diminished or abolished.

Strictly speaking there are no muscular paralyses or atrophies in trench foot. The phenomena of functional impotence are the consequence of pain, and after this acute stage they are due to a certain state of muscular meioprobia, doubtless dependent upon consistent vaso-motor troubles, which may increase at any time under the influence of cold and fatigue.

The subjective symptoms of trench foot are painful sensations, which never fail in this affection. The acute stage is very painful, but these pains occur also in a mild form, and there is quite a disproportion between the objective phenomena and the subjective sensations.

Even after recovery, quite a number of phenomena remain, such as, — a numbness of the feet, cramps in the calves, more or less characterized algesia of the lower limbs, which, with the circulatory and thermic troubles of the extremities, form the symptomatic complications of aeroparesthesia with frigosis, in which they may be witnessed aside from the actual condition of trench foot.

The complications of trench foot usually occur without a general or febrile reaction when there is no secondary infection. The latter breaks out especially in the serious forms. It is evident that necrosed tissues give exceptional opportunity for infection to all sorts of germs to be found on feet soiled with mud. Among the secondary infections are tetanus and gas gangrene, due to anaerobia.

The duration and prognosis of trench foot depend entirely upon the gravity of the disease. In the milder forms a patient could be considered as recovered after a few days, if vasomotor and painful complications did not immobilize him for several weeks. The more serious forms last much longer on account of the more important necrotic process, but the possible secondary mutilations and infections show what a serious prognosis those grave forms have.

*Pathology.*

At the beginning of the war, the name given to such a condition was that of frozen feet. Later it was decided that this name, which was justified by the aspect of the lesions, was not so by the climatic conditions of their apparition, for they occurred especially during damp weather, even when the thermometer did not fall below the temperature of melting ice. Then the question came up as to whether cold was one of the main factors causing such accidents. Several other explanations were given, such as, — compression due to wrapped leggings. That is why the name of trench foot was given, because it does not emphasize either the nature or the cause of the action. All these accidents are essentially due to a primitive ischemic state, which causes secondary nervous and trophic complications, and which results undoubtedly from the reflex vasoconstrictive action produced by the cold through the intermediary of the sympathetic system. These circulatory troubles occur in the lower as well as the upper limbs, even the face, and constitute the syndrome of " aero-paresthesia with frigosis " in men perfectly normal, but who have suffered from abnormally prolonged and intense cold. If this syndrome is seen in soldiers who have had frozen feet, it is not a mere coincidence. These two series of facts are closely united, for " aeroparesthesia with frigosis " is nothing but the first manifestation in the mildest form of a series of circulatory nervous and trophic complications due to cold, and of which frozen feet is a consequence.

It is easy to understand, therefore, why trench frost bite affects practically only the feet, and not always at a very low temperature. This is because the vasoconstrictive action of the cold is helped by the surrounding circumstances. One must first consider immersion in water and mud, for at a given low temperature refrigeration and loss of heat is much greater in a liquid than in a dry medium. Moreover, circulation is greatly hindered by immobility and prolonged vertical position. Dampness also causes a constriction of the shoes and leggings, and therefore a swelling of the lower extremities. Finally, all these factors act upon tissues whose vitality and resistance are certainly diminished in most of the cases on account of the pre-existent troubles caused by the " aero-paresthesia with frigosis".

Nothing differentiates trench foot from accidents of the same kind which occur away from the trenches, such as those which have been observed in soldiers sent back from Germany, who were obliged to take long trips sitting down in cold trains, in the middle of winter. Trench foot also resembles complications of the hands, to which aviators are subject. The name of trench foot has therefore a too limited meaning. A better name would be that of coldness of feet, as the word *frozen* does not agree with the meteorologic conditions.

